# The blind men and the AML elephant: can we feel the progress?

**DOI:** 10.1038/bcj.2016.33

**Published:** 2016-05-13

**Authors:** S Tauro

**Affiliations:** 1 Dundee Cancer Centre, Ninewells Hospital & Medical School, University of Dundee, Dundee, Scotland, UK

## Abstract

The pharmacological therapy of non-promyelocytic acute myeloid leukemia (AML) has remained unchanged for over 40 years with an anthracycline–cytarabine combination forming the backbone of induction treatments. Nevertheless, the survival of younger patients has increased due to improved management of the toxicity of therapies including stem cell transplantation. Older patients and those with infirmity that precludes treatment-intensification have, however, not benefited from improvements in supportive care and continue to experience poor outcomes. An increased understanding of the genomic heterogeneity of AML raises the possibility of treatment-stratification to improve prognosis. Thus, efforts to identify agents with non-conventional anti-leukemic effects have paralleled those aiming to optimize leukemia cell-kill with conventional chemotherapy, resulting in a number of randomized controlled trials (RCT). In the last 18 months, RCTs investigating the effects of vosaroxin, azacitidine and gemtuzumab ozogamycin and daunorubicin dose have been reported with some studies indicating a statistically significant survival benefit with the investigational agent compared with standard therapy and potentially, a new era in AML therapeutics. Given the increasing costs of cancer care, a review of these studies, with particular attention to the magnitude of clinical benefit with the newer agents would be useful, especially for physicians treating patients in single-payer health systems.

## Introduction

In the year 2016, it is sobering to reflect that the pharmacological treatment of most forms of acute myeloid leukemia (AML) has remained unchanged over four decades. The relatively limited therapeutic stratification of AML owes more to serendipitous interventions and observations than an enhanced understanding of disease biology or pharmacodynamics.^1, 2^ The improvement in remission rates of non-acute promyelocytic leukemia AML following induction chemotherapy too should be credited to progress in supportive care and not novel anti-leukemic strategies.^3^ Regrettably, most remissions remain short-lived, impacting particularly on patients over 60 years of age, in whom the disease is frequent and treatment-intensification often precluded by co-morbidity. Recent developments in the genomic and functional characterization of AML^4, 5^ along with the availability of a plethora of novel therapeutic options including small molecule drugs have promised much to improve outcomes over conventional therapy.

Attempts to translate promising *in vitro* and early-phase clinical studies of newer agents into routine hematology practice has begun in earnest through phase 3 randomized controlled trials (RCTs). Considered the gold standard to evaluate the effectiveness of interventions, RCT can be practice-changing, but an enthusiastic analysis of ever-increasing numbers of measures in trials lacking sufficient power could make RCT a double-edged sword, particularly in rarer diseases such as AML. Too often, data of elephantine proportions from contemporary RCTs are accorded the same dogmatic response as that from the proverbial blind men in their description of the elephant. In the fable, with just the powers of proprioception at their disposal to examine a limited part of the elephant's anatomy, the resulting irreconcilable descriptions of the animal could almost have been predicted. This study of the elephant would appear to be a truly blinded one with no obvious external confounders contributing to the conclusions; the financial, human, intellectual and time investment in RCTs, combined with expectations from clinicians and patients faced with a life-shortening illness and dearth of effective treatments, means that the desire to identify the next poster child in AML therapeutics is strong. RCTs reported recently thus appear to herald the arrival of treatment strategies including novel drugs, with the potential to improve survival in AML,^6, 7, 8, 9^ but in the interests of fairness to other ‘blind men', particularly those based in single-payer healthcare systems, a different perspective on the same data set could be useful.

## Vosaroxin

The phase 3, double-blind, placebo controlled trial, VALOR, randomized over 700 patients to investigate whether the addition of the quinolone derivative vosaroxin to cytarabine improves survival in relapsed or refractory AML.^6^ Unstratified analysis showed no survival difference between the arms of the study. Following adjustment for pre-randomization variables, a median survival difference of ~2 months in favor of the vosaroxin arm was observed, maintained in a predefined analysis of patients over 60 years old. Based on these results, how meaningful an advance is combination therapy with vosaroxin–cytarabine toward establishing a new standard of care in poor-risk AML? While complete remission (CR) in the vosaroxin arm was almost double that with standard therapy (30 vs 16% respectively; *P<*0.0001), one cannot help noticing that these figures appear inferior to those achieved in a previous RCT of high-risk AML (MRC AML-HR) where the CR rates approach 60%.^10^ These differences should nevertheless be interpreted with caution, given the heterogeneity in patient demographics and disease, as well as disease definition criteria in the two RCTs separated by a time-lapse of 10 years. When one examines early-mortality rates between the two arms of VALOR, these are undoubtedly similar. Worryingly, however, ∼80% of participants had to discontinue therapy after the first cycle (of an intended total of four cycles) for reasons of treatment failure, death or toxicity. That discontinuation rates in the experimental arm containing vosaroxin were similar to the control provides scant reassurance: if only 20% of the participants with a performance status of ⩽2 at trial entry are capable of proceeding with further therapy, then our definition of the ‘standard' requires re-evaluation. Based on the 1-year survival data in VALOR, 19 patients would need to be treated with cytarabine and vosaroxin to prevent one death after ‘standard' therapy. Had VALOR been sufficiently powered for the survival benefit of combination therapy in the over-60 s sub-analysis to be conclusive, many physicians in single-payer healthcare systems would still wonder whether the anticipated quality-of-life and resource utilization for a 2-month median survival benefit is sufficient for vosaroxin–cytarabine to merit consideration as a cost-effective, new standard.

## Azacitidine

In contrast to vosaroxin, the hypomethylating nucleoside analog azacitidine was manufactured in the 1960s, but its evaluation in RCTs for patients with myeloid malignancies has been more recent.^7, 11^ In patients with intermediate-2 and high-risk myelodysplastic syndromes (MDS) including chronic myelomonocytic leukemia (CMML) and AML with a marrow blast count of 20–30%, azacitidine has been shown to prolong survival by a median of 9.5 months over conventional care regimens (CCRs) including best supportive care (BSC), low-dose (non-intensive) cytarabine or intensive chemotherapy (hazard ratio 0.58, *P*=0.0001).^11^ No additional RCT has been undertaken to confirm this result but in conjunction with the manufacturer's patient access schemes, in the UK, azacitidine was considered a cost effective option to treat sub-groups of MDS, including CMML and AML patients grouped either through similarities of predicted clinical outcomes^12^ or of morphological features. The results of a subsequent phase 2 study indicating lack of significant benefit with azacitidine in CMML, as well as the inability of mutational and methylation profiles to stratify responses are therefore disappointing and highlight unresolved challenges to the prospective identification of patients that would optimize cost-effectiveness of the drug.^13^

Nevertheless, the improvement in outcomes in low-blast count AML patients treated with azacitidine^11^ mandated an investigation of the drug in patients with a higher blast percentage.^7^ Conducted over 18 countries, the AZA-AML-001 RCTs asked if azacitidine compares favorably to CCR. As with the previous study in MDS/AML,^11^ the selection of the CCR option (including BSC) for randomization against azacitidine was left to the discretion of the treating physicians, making the trial not completely randomized and potentially open to bias. This design may have a pragmatic basis: clinicians are known to be less enthusiastic about randomizing patients between intensive and non-intensive treatment,^14^ or older patients may be reluctant to participate to a trial involving more intensive interventions of, as yet, unproven benefit. However, the grouping of heterogeneous therapies known to alter patient outcomes^15^ under the umbrella of CCR means that the final analysis could have masked meaningful differences in outcomes between the individual CCR options and azacitidine. There are further confounding variables with the potential to influence the results: standards for BSC have not been explicitly defined, so a role for variation in inter-institutional clinical practice as a determinant of survival cannot be excluded.^16, 17^ Whether the frequency of contact between healthcare professionals and patients differed between the study arms to introduce bias also requires consideration.^18^ Unsurprisingly perhaps, the study failed to meet its primary end point as azacitidine therapy did not associate with a statistically significant improvement in overall survival. Through a series of sub-analysis, many of which were pre-specified but underpowered, a survival benefit with azacitidine over BSC was identified. In another comparison, the median survival until subsequent therapy (time to next therapy (TTNT)) too favored azacitidine over CCR by almost 5 months (hazard ratio=0.76, 95% confidence interval, 0.60–0.96; *P*=0.019). The choice of TTNT as an end point is an interesting, arguable one since its use should optimally be restricted to the analysis of outcomes in diseases with effective, life-prolonging sequential therapies^19^ unlike relapsed AML in the elderly, where there are few meaningful therapeutic options. Moreover, there are no prescribed criteria for specifying TTNT in AML, so the timing of therapy is governed by clinical judgement, an easy source of bias.

An often-cited merit of hypomethylating therapy is its perceived ability to improve survival in patients with poor-risk cytogenetics,^20, 21, 22, 23^ in whom low-dose cytarabine and intensive chemotherapy are ineffective as primary therapy. Indeed, the sub-analysis of patients with poor-risk karyotype in AZA-AML-001 lends further support to this perception, but the inadequate power of the study fails to make the results more definitive. Whether all elderly patients in whom performance status or co-morbidity precludes intensive chemotherapy, or just those with a poor-risk karyotype should receive azacitidine for an improvement in outcomes thus remains an open question.

## Gemtuzumab ozogamicin

Despite investigation in multiple RCTs, few drugs have demonstrated the capacity to divide opinions as much as Gemtuzumab ozogamicin (GO).^24, 25, 26, 27, 28^ Accelerated approval by the Food and Drug Administration (FDA) in 2000 for the therapy of relapsed AML in older patients unsuitable for other intensive therapies^29^ was followed by an amazing fall-from-grace with a follow-up SWOG RCT, indicating not only lack of benefit but also suggesting increased mortality with GO.^28^ A confounding role for the unexpectedly low mortality in the control group of the study, as well as anthracycline dosage differences between the cohorts continues to be debated, but the drug was voluntarily withdrawn from the market by the manufacturer in 2010. In combination with chemotherapy, GO has now been studied in further RCTs, some of which indicate a reduction in relapse risk and improvement in overall survival without increased toxicity.^24, 25, 27^ Meta-analyses of RCTs too have yielded conflicting conclusions regarding a survival benefit.^30, 31^ Patients in whom a consistent benefit with GO can be cautiously claimed through data from RCTs (including the SWOG study^28^) and meta-analyses are those with favorable cytogenetics with no apparent advantage to those with unfavorable karyotype.^30, 31^ Highlighting the polarization of opinion regarding GO, the drug is included in all induction schedules for newly diagnosed patients with good and intermediate cytogenetics in the UK-based AML18 and AML19 RCTs.

Amidst this fervour, is a recent report on a randomized comparison between GO and supportive care (including oral chemotherapy with hydroxycarbamide or nucleotide analogs) in AML patients unsuitable for intensive chemotherapy.^9^ Fractionated doses of GO (9 mg m^−^^2^) were administered on day 1 and day 8 as induction treatment, followed by 2 mg m^−^^2^ once a month for further eight cycles. Of the patients who received induction therapy, over half received at least one additional dose, with the median number of infusions being 3. Patients receiving GO had an assessment of disease response beginning at day +36 with just under a quarter achieving a response (CR 8.1% and CR incomplete 16.2%). ‘Stable disease' was observed in ~40% of patients receiving GO; whether this is a valid, clinically meaningful end point in AML cannot be ascertained since no data from the supportive care arm were provided. With the median survival in both groups being <6 months, a survival benefit of 1.3 months was observed in favor of GO (hazard ratio, 0.69; 95% confidence interval, 0.53 to 0.90; *P*=0.005), in the absence of a significant increase in toxicity particularly to the liver. Thus, while single-agent GO has demonstrable anti-leukemic effects and tolerability in patients ineligible for intensive chemotherapy, its cost-effectiveness over low-dose cytarabine associated with similar responses^15^ requires evaluation. The median time to CR/CR incomplete with GO (36.5 days, range 14–139)^9^ appears faster than with low-dose cytarabine (CR time 114 days, range 50–313)^15^ but comparative data on quality-of-life and resource utilization through a randomized trial of the two drugs do not currently exist. Attempts to augment the anti-leukemic effects of therapy with combination low-dose cytarabine and GO include RCTs in patients unsuitable for more intensive approaches, but despite a near-doubling of response rates with combination therapy compared with low-dose cytarabine there was no discernible survival advantage.^32^ Resource utilization (platelet and antibiotic support and in-patient care) was higher with combination therapy and the absence of quality-of-life data in patients achieving CR means that the global impact of achieving higher remission rates is uncertain. For these reasons, the status of low-dose cytarabine as the therapeutic standard for elderly or infirm AML patients remains unchallenged.

## High-dose daunorubicin

The quandary of the blind men with the pachyderm is well-illustrated not just in the interpretation of RCTs involving newer agents, but also in the evaluation of conventional chemotherapeutic dosage in newly diagnosed patients.^8, 33, 34^ Since most contemporary clinical studies in AML include drugs purportedly targeting leukemic cells, the assumption that half-a-century's experience with genotoxic chemotherapy would have optimized induction schedules for AML therapy is a reasonable one. However, results from recent studies on outcomes following higher doses of daunorubicin (90 mg m^−^^2^) compared with 45 mg m^−^^2^ or 60 mg m^−^^2^ during induction treatment with cyatarbine suggest otherwise,^8, 33, 34^ and indicate that adages from 1973 continue to be relevant in 2016. Indeed, ‘daunorubicin is obviously a difficult drug to handle since in an empiric way there is a narrow dosage range and schedule which will induce an optimal aplasia, destroying proliferating leukemia cells but sparing stem cells'.^35^

In an RCT involving 582 patients conducted by the Eastern Cooperative Oncology Group (ECOG) first reported in 2010,^33^ and recently updated,^34^ the CR rate with higher-dose daunorubicin (71%) was superior to that (57%) with a 45 mg m^−^^2^ dose and translated into a significant improvement in overall survival. However, using induction schedules containing 50 mg m^−^^2^ daunorubicin, the UK-based MRC trials had previously demonstrated outcomes that were comparable to those achieved with higher-dose daunorubicin.^36^ A randomized comparison of higher-dose daunorubicin with 60 mg m^−^^2^ was therefore undertaken in 1206 patients in the AML-17 study: although the follow-up period was relatively short, this failed to demonstrate the benefits with 90 mg m^−^^2^ over 60 mg m^−^^2^ daunorubicin either in CR rates or longer-term survival, but suggested increased mortality at 60 days with higher-dose daunorubicin.^8^ Could one therefore conclude that induction regimens containing 90 mg m^−^^2^ daunorubicin are superior to 45 mg m^−^^2^ but not 60 mg m^−^^2^ thereby establishing 60 mg m^−^^2^ as the standard of care? Attempts to address this question require comment on the design and drug dosage schedules of the two RCTs: induction therapy in the ECOG schedule involved administration of daunorubicin on 3 consecutive days and continuous infusion of cytarabine (100 mg m^−^^2^ per day for 7 days),^33, 34^ whereas in AML-17 a more ‘staggered' schedule of daunorubicin (days 1, 3 and 5) and cytarabine (200 mg m^−^^2^ per day in divided doses 12 h apart, for 10 days) was followed. Furthermore, in the North American study^3, 33, 34^ only patients not achieving clearance of blasts in a ‘nadir' biopsy following 12–14 days of therapy received additional doses of daunorubicin. In contrast, in the MRC study assessment of disease response occurred at a later stage (days 21–25), with most patients receiving a second daunorubicin-containing induction cycle (50 mg m^−^^2^ days 1, 3 and 5). Thus, the cumulative doses of daunorubicin administered to patients in CR who continued with this study (330 mg m^−^^2^ and 420 mg m^−^^2^ in each arm, respectively) exceeded that in the SWOG study, where over 90% of participants in remission received a total dosage of ⩽270 mg m^−^^2^. Only a minority of patients (<10%) within the higher-dose arm in the SWOG study, requiring two cycles of induction to attain CR, received 405 mg m^−^^2^ daunorubicin. Hence, the total daunorubicin exposure for a larger proportion of SWOG participants (including most in the ‘higher-dose' arm) was less than in the MRC study. Further differences that confound a direct comparison of the two studies include the provision of cytokine support, risk-stratification criteria and choice of consolidation therapy. It is therefore highly likely that in the foreseeable future, geographical location will continue to determine the design of induction schedules and doses of daunorubicin for newly diagnosed patients with AML.

There is no doubt that the RCTs reviewed here ask pertinent questions in areas of unmet medical need.^6, 7, 8, 9^ These multi-centric international trials demonstrate clearly, the desire among investigators to evaluate a hypothesis of interest despite the potential for logistical difficulties and variation in legislation relevant to research governance in different countries. As with all good science, however, the evidence requires to be examined more forensically before a transition to routine clinical practice can occur. In an era when new drugs are increasingly being priced high, it is no longer appropriate to focus the appraisal of newer agents or interventions exclusively to a statistically valid, clinical end point.^37, 38^ A health technology assessment (http://www.nets.nihr.ac.uk/programmes/hta) that incorporates the magnitude of benefit, along with social and ethical considerations is now indispensable for establishing cost-effectiveness, and to avoid any misplaced ‘shroud-waving' arising from negative decisions on funding of new technologies. The debate about anti-neoplastic drugs is more emotive than the therapy for many chronic diseases since there is a dearth of suitable therapies for many cancers and continuing concern surrounding the affordability of proven, life-prolonging drugs.^39^ To apportion all blame to the pharmaceutical industry for the spiraling costs of healthcare, however, would be inappropriate; while most physicians may be unaware of the nuances of health economic modeling, they have a responsibility to inform the cost-benefit dialog around novel treatments by identifying progress that is clinically, and not just statistically meaningful. With the recently described newer pharmacological agents and therapeutic strategies,^6, 7, 8, 9^ subsets of AML patients could experience an improvement in outcomes. As one waits impatiently for the scientific tools to specify these subsets prospectively, the premature adoption of newer treatments for managing all patients with AML, could be counter-productive. Rather than viewing the opinions expressed here as the ‘pouring of cold water' on novel data by another ‘blind man', this perspective should be considered as contributory to the recently published data sets. After all, had the blind men in the fable adopted a collaborative, non-dogmatic approach, it might have been possible to reach a fairly sound description of the elephant.^40^
